# Nedd4-Mediated Increase in HIV-1 Gag and Env Proteins and Immunity following DNA-Vaccination of BALB/c Mice

**DOI:** 10.1371/journal.pone.0091267

**Published:** 2014-03-10

**Authors:** Brad Lewis, Stephen Whitney, Lauren Hudacik, Lindsey Galmin, Maria Cecilia Huaman, Anthony D. Cristillo

**Affiliations:** Advanced BioScience Laboratories, Inc., Rockville, Maryland, United States of America; University of Cape Town, South Africa

## Abstract

The late assembly domain of many viruses is critical for budding. Within these domains, encoded in viral structural proteins, are the conserved motifs PTAP, PPxY and YPxL. These sequences are the key determinants for association of viral proteins with intracellular molecules such as Tsg101, Nedd4 and AIP1/ALIX. While roles for Tsg101 and AIP1/ALIX in HIV-1 budding have been well established, less is known about the role of Nedd4. Recent studies, however, have identified a function for Nedd4-like protein in HIV-1 release. In this study, we investigated post-transcriptional changes of Nedd4 following SHIV_SF162P3_ infection of rhesus macaques, its role on HIV-1 p24 and gp120 levels *in vitro* and its potential as an immune modulator in HIV vaccination of BALB/c mice. Increased Nedd4 protein levels were noted in both CD4^+^ and CD8^+^ T cells following SHIV_SF162P3_-infection of naïve macaques. Transient co-transfection studies in 293 cells with HXB2 and Nedd4 demonstrated a Nedd4-mediated increase in p24 and gp120 levels. This increase was found to be dependent on the Ca^2+^/calmodulin-regulated phospholipid binding C2 domain and not ubiquitin ligase activity or HIV LTR activity. Co-transfection of Nedd4 with plasmid DNA expressing Gag or Env was further shown to augment both intracellular and extracellular Gag or Env proteins. To assess the potential of Nedd4 as an immune modulator, BALB/c mice were immunized intramuscularly with plasmid DNA encoding HIV *gag*, *env* and Nedd4. Nedd4 co-administration was found to increase serum anti-p24 but not anti-gp120 antibodies. Nedd4 co-injection was found to have no affect on Gag- or Env-specific IFNγ but had a trend of increased Gag-specific IL-6, IL-17A and TNFα that was not seen following Env stimulation. Based on our initial findings, Nedd4-mediated changes in HIV protein levels and its potential use in HIV-1 vaccine development warrants further investigation.

## Introduction

The role of ubiquitin ligases on late stage viral processing and budding has been investigated by many laboratories working on different viruses including but not limited to human immunodeficiency virus-1 (HIV-1), feline immunodeficiency virus (FIV), Rous sarcoma virus (RSV), Ebola virus, Avian sarcoma virus and Murine leukemia virus (MLV) [1]–[5]. Structural proteins, such as HIV-1 Gag, have been found to be mono-ubiquitinated and depletion of intracellular-free ubiquitin pools, using proteasome inhibitors, shown to inhibit budding [6]–[8]. While many studies have been conducted in order to dissect the mechanism(s) by which this ubiquitination occurs and is regulated, the signaling pathways in host target cells that influence viral budding are not completely understood.

While most regions of HIV-1 Gag and other retroviral structural proteins appear to be dispensable for budding, an essential region has been identified as the late assembly (L-) domain [9]–[13]. The core element in the L-domain has been shown to include the following conserved sequences: PTAP, PPxY, LxxLF, and YPxL [10]–[12], [14], [15]. The core element has been found to be required for efficient pinching off of the virus bud [9], [11], [12]. Furthermore, expression of HIV-1, RSV or BLV Gag L-domain deletion mutants resulted in a budding defect in which host cells remained covered with viral particles tethered to the membrane [7], [8]. The L-domain core sequences (PTAP, PPxY, LxxLF and YPxL) are well conserved throughout the retroviridae family and thus support their importance in viral budding and pathogenesis.

Ongoing studies seeking to bridge the gap “mechanistically” between viral budding, ubiquitination and the L-domain core element have identified class E vacuolar protein sorting factors, tumor susceptibility gene product (Tsg101) [16]–[18] and AIP1/ALIX [1], as key mediators of HIV-1 Gag trafficking and viral budding. Tsg101, an ESCRT-I (endosomal sorting complex required for transport I) component, has been shown to interact with the PTAP motif of HIV-1 Gag p6 [19], [20] and mediate budding via multivesicular bodies [21], [22]. Studies have clearly demonstrated, using a dominant negative Tsg101 [23]–[27] or Tsg101-targeted siRNA [16], that this ESCRT-I component is critical for HIV-1 budding. AIP1/ALIX has also been shown to play a key role by binding to the YPxL motif of HIV-1 Gag and thereby associating HIV-1 Gag and Tsg101 to the endosomal complex ESCRT-III. Conversely, it has been shown that viruses of the “PPxY” L-domain type such as HTLV-I, RSV and Ebola virus utilize Nedd4 (E3 ubiquitin ligase) family members to mediate viral trafficking and budding [17], [28]–[36].

While the role of Nedd4 on viral egress for “PPxY” type viruses has been well studied, several laboratories in recent years have elucidated a role for Nedd4 and Nedd4 family members on viruses containing PTAP and YPxL motifs. To this end, the Nedd4-like (Nedd4L) protein was shown to rescue HIV-1 budding defects caused by a lack of Tsg101- and ALIX late domains [5]. Nedd4 has also been shown to be recruited by ALIX in facilitating HIV-1 budding via ALIX-dependent ubiquitination [37]. Furthermore, exogenous expression of Nedd4-2 s was found to increase HIV-1 budding that was shown to be dependent on ubiquitination [38] and the truncated C2 domain [39].

We here identify increased Nedd4 protein levels in both CD4^+^ and CD8^+^ T cells following SHIV_SF162P3_-infection of naïve macaques. Transient co-transfection of 293 cells with HIV-1_HXB2_ and Nedd4 subsequently demonstrated a Nedd4-mediated increase in p24 and gp120 levels that was dependent on the Ca^2+^/calmodulin-regulated phospholipid binding C2 domain but not ubiquitin ligase activity. Vaccination of BALB/c mice with Nedd4 co-administration yielded increase serum anti-p24 but not anti-gp120 antibodies. Nedd4 co-injection was found to have no affect on Gag- or Env-specific IFNγ but had a trend of increased Gag-specific IL-6, IL-17A and TNFα that was not seen following Env stimulation.

## Materials and Methods

### Housing and Care of Rhesus Macaques

The animals in this study were Indian rhesus macaques (*Macaca mulatta*) and were housed at the Advanced BioScience Laboratories, Inc. (ABL) animal facility. All animals were cared for and procedures performed under a protocol approved by the ABL Animal Care and Use Committee (animal welfare assurance no. A3467-01; protocol no. AUP308). Furthermore, the macaques in this study were managed according to the animal husbandry program of the ABL Animal Facility, which aims at providing consistent and excellent care to nonhuman primates at the vivarium. This program operates based on the laws, regulations, and guidelines promulgated by the United States Department of Agriculture (e.g., the Animal Welfare Act and its regulations, and the Animal Care Policy Manual), Institute for Laboratory Animal Research (e.g., Guide for the Care and Use of Laboratory Animals, 8th edition), Public Health Service, National Research Council, Centers for Disease Control, and the Association for Assessment and Accreditation of Laboratory Animal Care (AAALAC) International.

The nutritional plan utilized by the ABL Animal Facility consisted of twice daily feeding of Labdiet 5045 High Protein Primate Diet and food intake was closely monitored by Animal Research Technicians. This diet was also supplemented with a variety of fruits, vegetables, and other edible objects as part of the environmental enrichment program established by the Veterinary staff and enrichment Technician. Pairing of animals as part of the environmental enrichment program was managed by the enrichment technician. All primary enclosures and animal rooms were cleaned daily with water and sanitized at least once every two weeks. Viral challenges were performed under anesthesia (Ketamine administered at 10 mg/kg) and all efforts were made to minimize suffering. None of the animals were euthanized as part of this study.

ABL’s routine health surveillance consists of physical examinations, tuberculin (TB) tests as well as clinical tests and observations. Animals are periodically screened for viruses and pathogens such as STLV, SRV, measles and herpes B virus, and SIV using serological and sensitive real time PCR-based assays developed at ABL. Animal observations and general health checks will be performed twice daily, seven days a week, on all animals assigned to this program. Daily observations of all animals will be done to assess their health and well being. Animals are observed for changes in stool condition, food consumption, evidence of trauma, signs of pain or distress, and the appearance of any clinical signs that indicate ill health. ABL has an established environmental enrichment plan for nonhuman primates compliant with the Animal Welfare Act. This enrichment plan includes social interaction through group housing, sensory and cognitive enrichment, and identification of and individualized treatment for psychological distress.

The program veterinarian is authorized to make decisions as to whether animals meet criteria that constitute humane endpoints that will result in removal of animals from study. The program veterinarian is in close communication with the principal investigator regarding the health status and medical conditions of animals enrolled in studies. SHIV-infected animals were used for this study and SHIV-infected animals may develop AIDS like syndromes, such as market drop in blood CD4+ T cells, severe weight loss, diarrhea and may acquire opportunistic infections. Under such circumstances these animals may be euthanized following the veterinarian’s recommendation.

### Virus Challenge of Rhesus Macaques

Four male, juvenile, naïve Indian origin rhesus macaques (L861, L866, L867, L868) were challenged intra-rectally with a single, high dose (TCID in rhesus PBMC: 1.02×10^4^/ml) concentration of SHIV_SF162P3_.

### Plasma Viremia

Animals were bled periodically following challenge and plasma viral load was assessed using a sensitive real time nucleic acid sequence-based amplification assay (NASBA) to quantitate SIV RNA [40].

### CD4 T CELL Immunophenotyping

Following challenge of rhesus macaques, animals were bled periodically and absolute CD4 T cell measurements were performed using the BD Biosciences TruCount™ platform using an immunophenotyping panel consisting of anti-CD3/anti-CD4/anti-CD8/anti-CD45 antibodies (BD Biosciences, San Diego, CA).

### Intracellular Detection of Nedd4 by Flow Cytometry

Intracellular levels of Nedd4 were measured by cytometry in rhesus macaque PBMC. Briefly, cells were pelleted by centrifugation at 1100×g for 5 min and were then resuspended in 100 µl FACS wash buffer (BD Biosciences) and stained with a cocktail of fluorochrome conjugated anti-CD3, anti-CD4 and anti-CD8 antibodies (BD Biosciences) at room temperature for 30 min. Cells were then washed with FACS wash buffer and resuspended in 250 µl Cytofix/Cytoperm buffer (BD Biosciences) for 15 min at 4°C. Following incubation, 2 ml of Perm/Wash buffer (BD Biosciences) was added to cells. Cells were pelleted by centrifugation and then resuspended in Perm/Wash buffer containing a rabbit anti-Nedd4 antibody (1∶100; Upstate, Charlottesville, VA). Cells were incubated for 15 min at 4°C, washed with 2 ml of Perm/Wash buffer followed by centrifugation and staining with a secondary goat anti-rabbit-RPE antibody (1∶50; Southern Biotech, Birmingham, AL) for 15 min at 4°C. Cells were washed with 2 ml of Perm/Wash buffer, centrifuged and then resuspended in 200 µl of FACS wash buffer (BD Biosciences). Acquisition of cells was performed by cytometry using a FACScalibur™ (BD Biosciences).

### Constructs and Molecular Clones

The HIV-1 molecular clone, HXB2, was a generous gift from Dr. Marvin Reitz (Institute of Human Virology, University of Maryland Biotechnology Institute). Human Nedd4 and Nedd4-2 plasmid DNA were generously provided by Dr. Hughes Abriel (Institute of Pharmacology and Toxicology, University of Lausanne, Lausanne, Switzerland) and rat Nedd4 (rNedd4) and Nedd4CSmut (rNedd4Csmut) plasmid DNA were gifts from Dr. Daniela Rotin (University of Toronto, Toronto, Canada). The hNedd4C2mut plasmid was generated by digestion of the hNedd4 construct with PpMuI and NdeI restriction enzymes (New England Biolabs, Ipswich, MA). Following isolation of the 1134 bp digested fragment, using the QIAquick Gel Extraction kit (Qiagen, Valencia, CA), PCR was performed using the following primers: Nedd4C2mut-F:5′GTACATCAAGTGTATCATATGCCAAGTACGCCCCCTAT-3′; Nedd4C2mut-R:5′-ATAGGTCCTTCCAAGGAGCCCGAACACCTCCACCGC-3′. The resulting PCR product (549 bp) was digested with PpMuI and NdeI restriction enzymes and subcloned into the PpMuI/NdeI-digested hNedd4 plasmid. Successful subcloning was verified by restriction digestion and sequencing. Corresponding empty plasmids for hNedd4, hNedd4-2, rNedd4 and rNedd4CSmut were used as controls in addition to the empty pEGFP construct.

### Transient Transfections

293 cells (ATCC, Manassas, VA) were transiently transfected using Lipofectamine 2000 (Life Technologies, Carlsbad, CA) according to the manufacturer’s recommended protocol. Briefly, 293 cells (6×10^6^) were suspended in 12 ml of RPMI-1640 (Quality Biological, Gaithersburg, MD), supplemented with 10% heat-inactivated fetal calf serum (FCS) (HyClone, Logan, Utah), 2 mM L-glutamine (Quality Biological), and 50 µM 2-mercaptoethanol (Sigma, St. Louis, MO), termed antibiotic-free RPMI-1640. Cells were then transferred to a 100 mm tissue culture dish and incubated for 24 hr at 37°C with 5% CO_2_. On the day of transfection, 60 µl of Lipofectamine 2000 was diluted with Opti-Mem I Reduced Serum Medium (Life Technologies) to give a final volume of 1.5 ml and incubated for 5 min at room temperature. A total of 24 µg DNA (i.e., 12 µg of HXB2 plus 12 µg of hNedd4 plasmid) was diluted in Opti-Mem I Reduced Serum Medium to give a final volume of 1.5 ml. The diluted DNA mixture was added to the Lipofectamine mixture and incubated at room temperature for 20 min. The DNA/Lipofectamine mixture (3 ml) was then added to the 100 mm culture of 293 cells and incubated for 24 hr at 37°C with 5% CO_2_. After 24 hr, the medium was replaced with 15 ml of RPMI 1640 supplemented with 10% heat-inactivated FCS, 2 mM L-glutamine, 50 µM 2-mercaptoethanol, 100 U/ml penicillin (MediaTech, Herndon, VA) and 100 µg/ml streptomycin (MediaTech), termed 10% cRPMI-1640. At 24, 48 or 72 hr, cells and cell supernatants were collected by centrifugation at 1100×g for 5 min. For siRNA transfection studies, 293 cells (0.8×10^6^ cells) were transiently transfected in 6-well tissue culture dishes, using Lipofectamine 2000 as outlined above, with HXB2 (0.75 µg) and siRNA (4 µg; Qiagen) generated against:

Nedd4 (5′-TAGAGCCTGGCTGGGTTGTTTTG-3′) or control GFP (5′-GCACAAGCTGGAGTACAACTACA-3′).

### HIV-1 p24 and gp120 Antigen Capture Assays

HIV-1 p24 and gp120 antigen capture ELISAs (Advanced BioScience Laboratories, Kensington, MD) were used to quantify supernatant and intracellular HIV-1 p24 and gp120, respectively, as recommended by the manufacturer and previously described [41].

### Radioimmunoprecipitation Assay (RIPA)

At 32 hr post-transfection, HXB2-transfected 293 cells with or without hNedd4 co-transfection, were labeled overnight with 35S-Met (250 µCi) in Methionine free DMEM (without L-glutamine, L-Met, L-Cys; Invitrogen) supplemented with 2% FBS (heat-inactivated and dialyzed), 2 mM L-glutamine (Quality Biological), 1 mM sodium pyruvate (Quality Biological) and 4 µM L-Cys (Sigma, St. Louis, MO) at 37°C with 5% CO_2_. After labeling, media was centrifuged at 1100×g for 5 min at room temperature. The clarified media was added to PBS-TD lysis buffer (final concentration: 1X PBS, 0.5% Triton X-100 (Biorad), 1% Deoxycholic acid (Sigma), supplemented with 1 µl/ml Protease Inhibitor cocktail (Sigma; containing pepstatinA, E-64, bestatin, leupeptin, aprotinin and 4-(2-aminoethyl) benzenesulfonyl fluoride) followed by incubation at room temperature for 30 min. Cells were lysed in PBS-TD lysis buffer supplemented with Protease Inhibitor cocktail (1 µl/ml) and benzonase (1 µl/ml Novagen; Madison, WI) and incubated for 30 min at room temperature. Cell lysates and media were precleared by incubation with normal human serum and protein-A sepharose (PAS; GE Healthcare). HIV-1 proteins were immunoprecipitated from precleared cell lysates and mediae with PAS and anti-HIV(+) human serum (Sera Care Life Sciences Inc., Oceanside, CA). Uninfected (HIV-1(−)) normal human serum was used as a control (data not shown). After a 2 hr immunoprecipitation at room temperature, samples were washed with PBS-TD buffer, resuspended in 1X Leammli sample buffer, boiled for 2 min and centrifuged at 1100×g for 1 min to clarify samples. Proteins were resolved by 10% SDS-PAGE. Gels were incubated for 45 min in 30% methanol and 7% acetic acid and incubated for 30 min in Amplify buffer (GE Healthcare). Gels were dried to filter paper, under vacuum, for 2 hr at 80°C and exposed to film for 24 hr at –70°C.

### Immunoprecipitations/Western Blot Analysis

293 cells were transiently transfected, as described above, with HXB2 alone or in combination with hNedd4, hNedd4C2mut, hNedd4-2, rNedd4 or rNedd4Csmut plasmid DNA. Following transfection (48 hr), cells were harvested by centrifugation at 1100×g for 5 min at 4°C, washed once with 1X PBS and resuspended at a concentration of 2×10^6^ cells/ml in lysis buffer A [(1% NP-40 (Sigma), 0.15 M NaCl, 25 mM Tris pH 7.5, 1 mM EDTA, 1 mM PMSF, 10 µg/ml leupeptin, 10 µg/ml aprotinin, 1 µM sodium orthovanadate (Sigma)]. Cells were incubated on ice for 20 min and then centrifuged at 17,530×g for 10 min at 4°C. The detergent soluble fraction (30 µl of supernatant) was mixed with an equal volume (30 µl) of 2X Laemmli sample buffer whereas the detergent insoluble fraction was resuspended in 1X Laemmli sample buffer. Samples were heated for 5 min at 95°C and proteins were separated by 10% SDS-PAGE (Protogel, National Diagnostics, Atlanta, GA). The remaining detergent soluble fractions (cell supernatants) were incubated with either a rabbit anti-GFP polyclonal antibody (Clontech, Mountain View, CA) or anti-Nedd4 monoclonal antibody (BD Biosciences) for 2 hr at 4°C, after which, protein A agarose (Santa Cruz Biotechnologies) was added and samples were incubated for an additional 30 min at 4°C. Samples were washed three times in buffer B (1% NP-40, 0.15 M NaCl, 25 mM Tris pH 7.5, 1 mM EDTA, 1 µM sodium orthovanadate), resuspended in 1X Laemmli sample buffer and heated for 5 min at 95°C. Proteins were separated by 10% SDS-PAGE and transferred to polyvinylidene difluoride (PVDF) membranes (Millipore, Bedford, MA), immunoblotted with either an anti-GFP monoclonal antibody (Clontech), rabbit anti-mouse Nedd4 polyclonal antibody (BD Biosciences) and detected by enhanced chemiluminesence (ECL) (GE Healthcare) according to the manufacturer’s instructions.

### Transient Transfection and Luciferase Report Assays

Transient transfection of Jurkat T cells was performed by electroporation using the Gene Pulser (Biorad, Hercules, CA) according to the manufacturer’s instructions. The LTR-luc Firefly luciferase reporter construct was co-transfected with the hNedd4 construct and a reporter vector that contains a cDNA encoding Renilla luciferase (pRL-TK) under the control of the herpes simplex virus thymidine kinase promoter (Promega, Madison, WI). pRL-TK was used to control for transfection efficiency. Jurkat T cells (10^7^ cells) were transfected with 10 µg of LTR-luc, 15 µg of hNedd4 and 0.25 µg of pRL-TK by electroporation (270V and 960 µF). Cells were incubated for 24 hr at 37°C, 5% CO2 in air and then stimulated with phorbol 12-myristate-13-acetate (PMA; Calbiochem, La Jolla, CA) and Ionomycin (Iono; Calbiochem, La Jolla, CA) as indicated. The Dual Luciferase assay (Promega, Madison, WI) was performed to determine both Firefly and Renilla luciferase activities in cell lysates. Briefly, stimulated cell suspensions were transferred to Eppendorf tubes and pelleted by centrifugation at 500×g for 5 min. Cell pellets were washed once with 1X PBS and then lysed with 50 µl of 1X Promega passive lysis buffer. Samples were vortexed for 30 sec, incubated at room temperature for 15 min and pelleted again for 5 min at 20,000×g. The luminescence of 100 µl of luciferase assay reagent added to 20 µl of each lysate was recorded using a Centro LB960 luminometer (EG&G Berthold, Gaithersburg, MD). Finally, 100 µl of Stop & GloTM reagent was added to the sample and a second luminescence reading recorded (Renilla luciferase).

### Antigens

The codon optimized gene coding for HIV-1_Ba-L_ gp120 were generous gifts from Dr. Marvin Reitz (Institute of Human Virology, University of Maryland Biotechnology Institute). Mosaic HIV-1 Gag immunogens (Clades A, B and C) were designed as described [42], [43] using the Mosaic Vaccine Designer software (http://www.hiv.lanl.gov/content/sequence/MOSAIC/makeVaccine.html). The selected mosaic *gag* sequences were based on both optimal 9-mer coverage and the breadth of predicted epitope responses as described [44] using the BIMAS software tool (http://www-bimas.cit.nih.gov/cgi-bin/molbio/ken_parker_comboform). Plasmid DNA used for mice immunizations, encoding clade A, B and C *gag* sequences, was under the control of a CMV promoter. Plasmid DNA expressing HIV-1_Ba-L_ envelope was used for immunization of mice as previously described [45].

### Mice Immunizations

BALB/c mice (n = 5 per group; 5–6 week old females; Taconic, Hudson, New York) were immunized at weeks 0, 2 and 4 by intramuscular injection with plasmid DNA encoding human Nedd4 (100 µg) alone (Group 1), HIV-1 *gag* (pool of 100 µg each of Mosaic Clade A, B, C)+HIV-1_Ba-L_ *env* (100 µg) (Group 2) or human Nedd4 (100 µg)+HIV-1 *gag* (pool of 100 µg each of Mosaic Clade A, B, C)+HIV-1_Ba-L_ *env* (100 µg) (Group 3). Group 4 mice (n = 3) were left un-immunized and served as naïve controls. Two weeks following the final immunization (week 6), mice were sacrificed, splenocytes and serum collected and serum antibody responses and splenocyte T-cell responses were performed. Typically, the immunological responses are minimal and are often undetectable in naïve, control animals. All animal studies were reviewed and approved by the Institutional Animal Care and Use Committee (IACUC) at Advanced BioScience Laboratories.

### Peptides

Gag peptides, used for *ex vivo* stimulation of splenocytes, were obtained through the NIH AIDS Reagent Program, Division of AIDS, NIAID, NIH: HIV-1 Consensus A Gag (15-mer) Peptides - Complete Set; HIV-1 Consensus B Gag (15-mer) Peptides - Complete Set; HIV-1 Consensus C Gag (15-mer) Peptides - Complete Set. For each clade, Gag peptides were resuspended in one peptide pool and used for *ex vivo* stimulation at a final per peptide concentration of 1 µg/ml. For Clade B Env (HIV-1_Ba-L_), 79 peptides (15-mers) with 11 amino acid overlapping residues were synthesized that comprise the gp120 Env protein sequence. Clade B Env (HIV-1_Ba-L_) peptides were resuspended in one peptide pool and used for stimulation at a final per peptide concentration of 1 µg/ml.

### Binding Antibody Assay

Serum samples were tested for Env-specific antibodies and Gag-specific antibodies using an enzyme-linked immunosorbent assay (ELISA) as previously described [45]. Antibodies were detected against HIV-1 p24 Gag or HIV-1_Ba-L_ gp120 proteins. Serum titers were determined as the highest dilution of immune serum producing ELISA values (A_450_ nm) greater than or equal to two times the binding detected with a corresponding dilution of pre-immune serum. Protein boost-mediated fold increase in ELISA titers was calculated as follows: ELISA Titers (DNA+Protein)/ELISA Titers (DNA). As expected, serum from naïve animals had no reactivity with Env and Gag antigens in the ELISA (data not shown).

### Murine IFNγ ELISPOT

The IFNγ ELISPOT assay was performed using murine splenocytes according to the manufacturer’s protocol (UCyTech, Netherlands) as previously described [45], [46].

### Cytometric Bead Array

Cytometric bead array (BD Biosciences, San Diego, CA) was performed to quantitate secreted Th1 (IFNγ, IL-2, TNFα), Th2 (IL-4, IL-5, IL-6) and Th17 (IL-17) cytokines from supernatants of peptide-stimulated murine splenocytes as described [46], [47].

### Statistical Analysis

Statistical significance of Nedd4-induced HIV-1 p24 or gp120 levels, *in vitro*, was demonstrated using a one way ANOVA followed by Tukey’s multiple comparison test. Statistical significance of Nedd4-induced vaccine-specific immune responses, *in vivo*, was demonstrated using the Kruskal-Wallis nonparametric test followed by Dunn’s multiple comparison test.

## Results

### Increased Nedd4 Protein Levels Following SHIV_ SF162P3_-Infection of Rhesus Macaques

**Figure 1:**
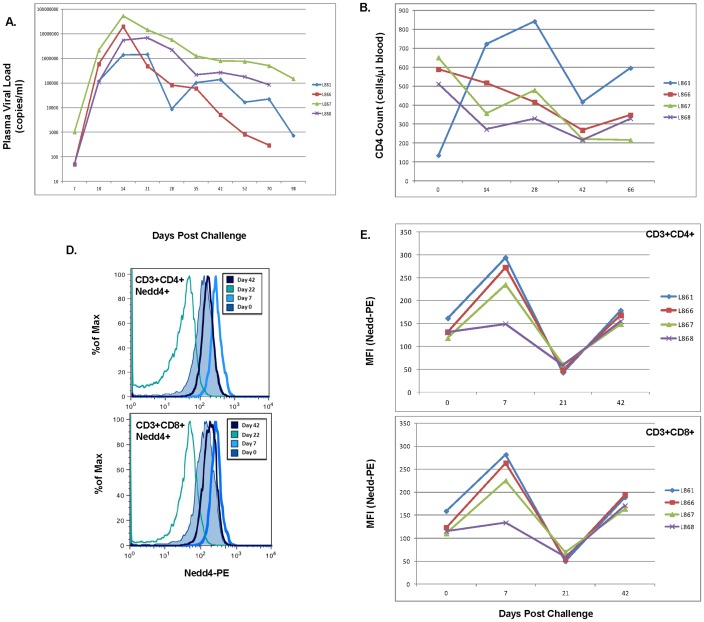
Increased Nedd4 Protein Expression Following SHIV_SF162P3_ Infection of Rhesus Macaques. Rhesus macaques were challenged intra-rectally with SHIV_162P3_. Plasma viral RNA load (A) was measured, using Real Time NASBA, at days 7, 10, 14, 21, 28, 35, 42, 52, 70 and 98 post-challenge and CD4 T cell counts (B) were quantified at days 0, 14, 28, 42, and 66 post challenge as outlined in the Materials and Methods (B). Intracellular staining of Nedd4 was performed using macaque peripheral blood mononuclear cells (D, E). Cells were acquired using an LSRII cytometer (BD Biosciences) and histogram representations (D) as well as graphical representations (E) are shown. Mean Fluorescence Intensities (MFI) of Nedd4^+^CD4^+^ T cells (E, upper panel) and Nedd4^+^CD8^+^ T cells (E, lower panel) are graphically represented for days 0, 7, 21 and 42 post-challenge.

Changes in intracellular Nedd4 protein expression were initially evaluated post-intra-rectal challenge of rhesus macaques with SHIV_SF162P3_. Nedd4 protein levels were assayed, using flow cytometry, following mucosal challenge of naïve rhesus macaques using a single, high dose concentration of virus. Following challenge, plasma viral RNA load was found to increase with peak viremia noted at day 14 post-challenge (Figure 1A). Absolute CD4 T cell counts were quantified at days 0, 14, 28, 42, and 66 post-challenge and were found to decrease in 3 of 4 macaques post-challenge (Figure 1B). By contrast, macaque L861 showed an increase in CD4 T cell counts post-challenge. Intracellular staining of Nedd4 using peripheral blood mononuclear cells was performed and both histogram representations (Figure 1D) as well as graphical representations (Figure 1E) are shown for Nedd4 expression in CD4 and CD8 T cells. Mean Fluorescence Intensities (MFI) of Nedd4^+^ in CD4^+^ (Figure 1E, upper panel) and CD8^+^ (Figure 1E, lower panel) T cells revealed an increase in Nedd4 protein levels at day 7 post infection followed by a decline by day 21 and then an increase by day 42.

### Increased Levels of Extracellular and Intracellular HIV-1 Gag and Env by Nedd4

**Figure 2:**
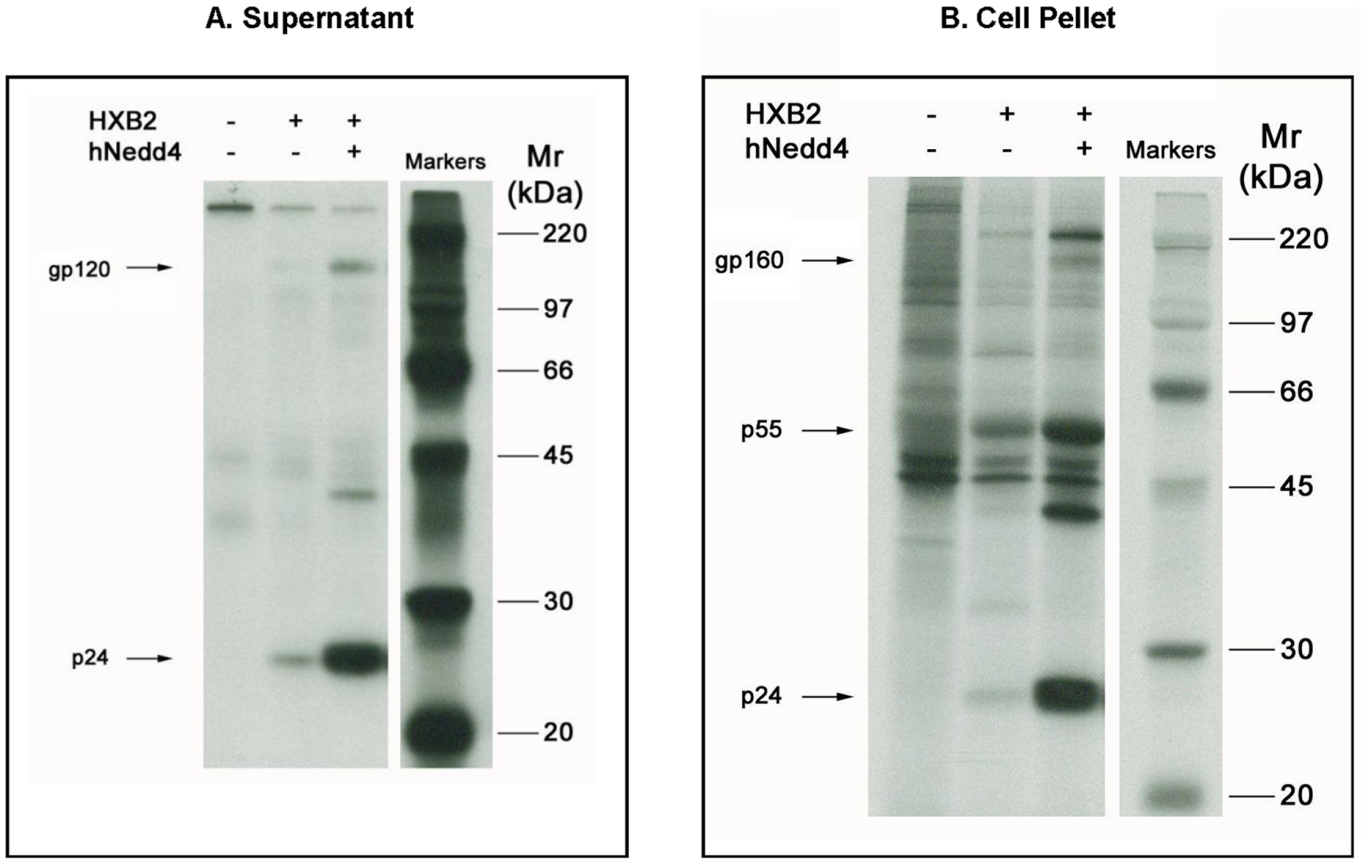
Increased Levels of Extracellular and Intracellular HIV-1 Gag and Env Proteins by co-expression of Nedd4. 293 cells were transiently transfected with HXB2 and a plasmid encoding human Nedd4 (hNedd4) as described (Materials and Methods). At 32 hr post-transfection, a radio-immunoprecipitation assay (RIPA) was performed by labeling cells for 12.5 hr with ^35^S-Met, immunoprecipitating target proteins using anti-HIV^+^ human serum, and resolving bands from cell media (A) and lysates (B) using 10% SDS-PAGE and autoradiography at –70°C. Bands corresponding to HIV-1 gp160, gp120, p55 and p24 proteins are shown.

Given the increased Nedd4 protein levels noted following SHIV infection of rhesus macaques, transient transfection assays were performed to assess a potential role for Nedd4 in viral replication as determined by HIV-1 p24 Gag and/or gp120/gp160 production. Using a radio-immunoprecipitation assay, it was found that 293 cells co-transfected with plasmid DNA expressing Nedd4 and the HIV-1 molecular clone, HXB2, yielded increased levels of secreted (Figure 2A) and intracellular p24 Gag compared to HXB2 alone (Figure 2B). Similarly, Nedd4/HXB2 co-transfection augmented secreted gp120 (Figure 2A) and intracellular gp160 (Figure 2B) compared to HXB2 alone.

### Increased HIV-1 p24 in Cell Supernatants by Nedd4 is Dependent on Ca^2+^/Calmodulin-Regulated Phospholipid Binding C2 Domain and Not Ubiquitin Ligase Activity

**Figure 3:**
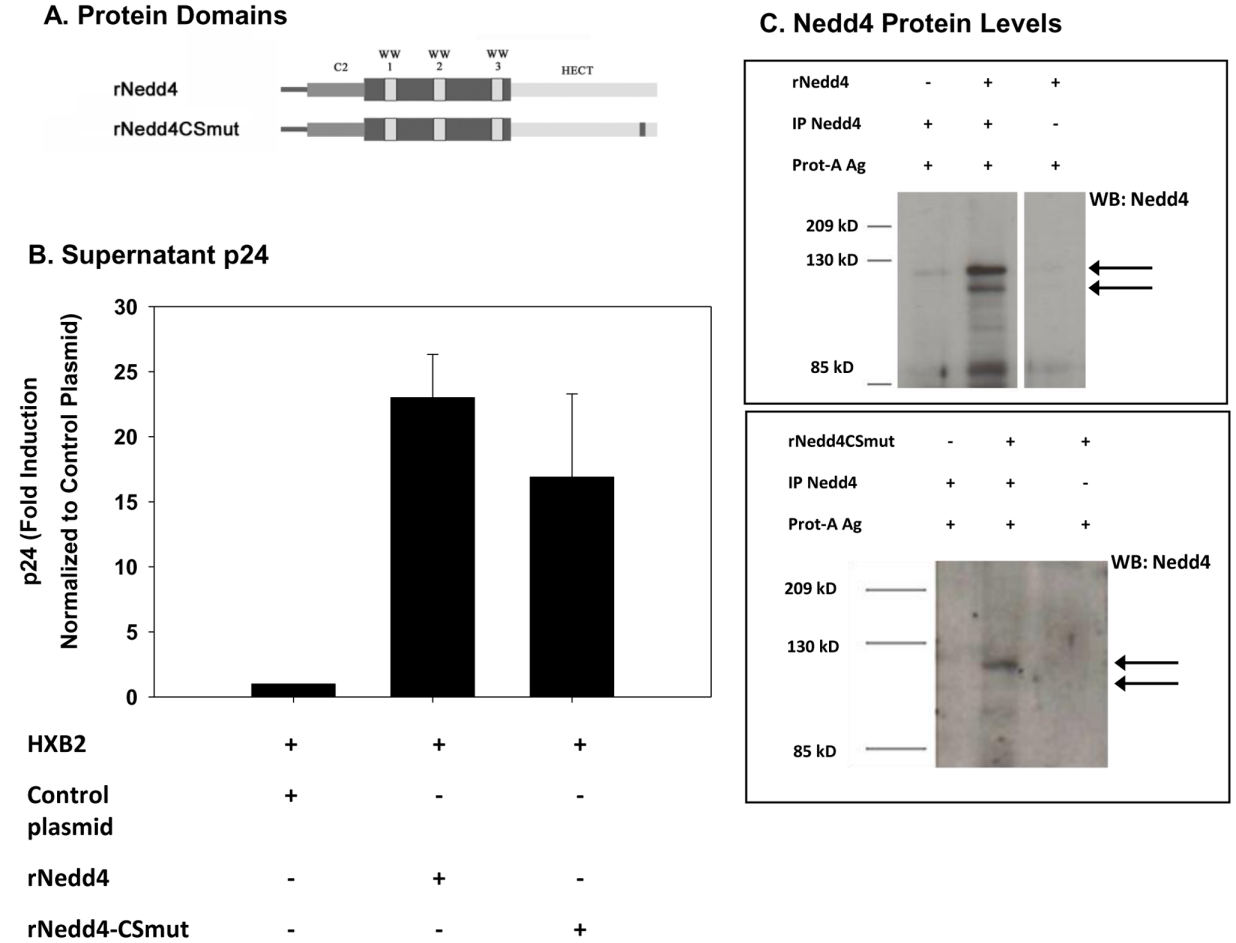
Ectopic expression of Nedd4 augments supernatant HIV-1 p24 levels Independent of HECT Domain Function. 293 cells were transfected with HXB2 and a plasmid encoding either rat Nedd4 (rNedd4) or a rat Nedd4 catalytic domain mutant (rNedd4CSmut) and at 48 hr post-transfection, cell supernatants were collected and p24 assays were performed as described (Material and Methods). Schematics highlighting the Ca^2+^/Calmodulin-regulated phospholipid binding domain (C2), WW protein binding domains (WW1–3) and E3 catalytic domain (HECT) for rNedd4 and rNedd4CSmut are shown (A). Mean p24± standard error values, calculated from data obtained from transfections conducted with rNedd4 (n = 7) and rNedd4CSmut (n = 5), are graphically represented (B). Statistical significance was shown for the increased p24 levels by rNedd4 (rNedd4 vs control at 48 hr: p<0.01; rNedd4CSmut vs control at 48 hr; p<0.01) using a one way ANOVA followed by Tukey’s multiple comparison test. No statistical difference was shown for rNedd4CSmut compared to rNedd4 at 48 hr (p>0.05) using this analysis. Immunoblot analysis, using a rabbit anti-mouse Nedd4 antibody, was performed on detergent soluble cell fractions as described (Materials and Methods). Nedd4 and Nedd4CSmut protein bands are indicated (arrows) (C).

**Figure 4:**
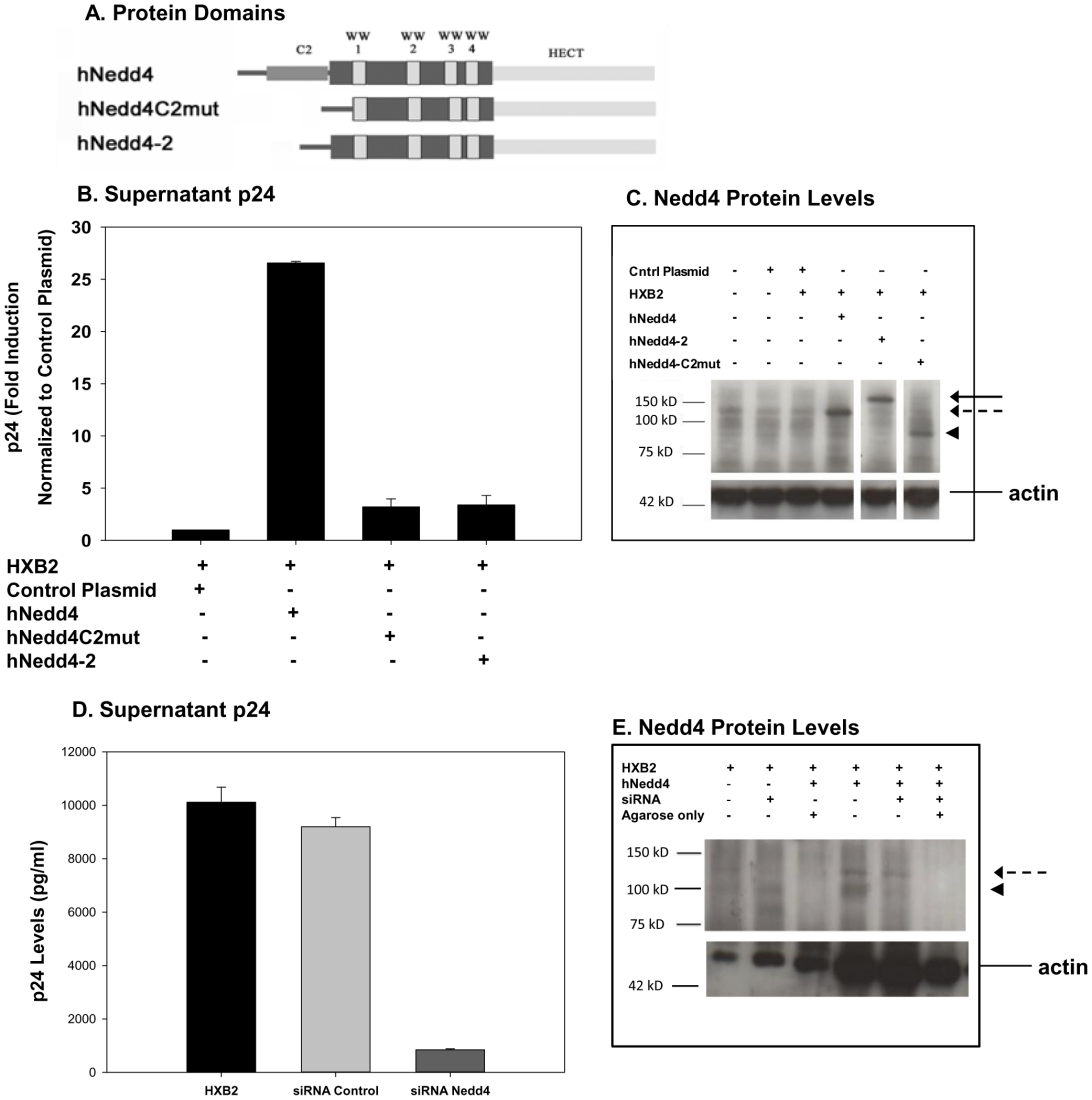
Increased p24 induced by Nedd4 Mediated by C2 domain. 293 cells were transiently transfected with HXB2 and a plasmid encoding human Nedd4 (hNedd4), human Nedd4 C2 deletion mutant (hNedd4C2mut) or a human Nedd4-2 splice variant that lacks the C2 domain (hNedd4-2) as described (Materials and Methods). Cell supernatants were collected at 48 hr post-transfection and p24 assays were performed. A schematic highlighting the Ca^2+^/Calmodulin-regulated phospholipid binding domain (C2), WW protein binding domains (WW1–4) and E3 catalytic domain (HECT) for hNedd4 is shown (A). Mean p24± standard error values were calculated from data obtained from transfections conducted with hNedd4 (n = 3), hNedd4C2mut (n = 3) and hNedd4-2 (n = 4) and are graphically represented (B). Statistical significance was shown for the increased p24 levels by hNedd4 (hNedd4 vs control at 48 hr: p<0.01) using a one way ANOVA followed by Tukey’s multiple comparison test. Statistical significance was not seen for hNedd4C2mut or hNedd4-2 (hNedd4C2mut or hNedd4-2 vs control at 48 hr: p>0.05). Western blot analyses, using a rabbit anti-mouse Nedd4 antibody, was performed to measure Nedd4, Nedd-2 and Nedd4-C2mut protein levels post-transfection as described (Materials and Methods). Nedd4 (dotted arrow), Nedd-2 (arrow), Nedd4-C2mut (arrow head) and actin protein bands are indicated (C). For siRNA experiments, 293 cells were transiently transfected with HXB2 alone or HXB2 with either Nedd4-targeted siRNA or irrelevant control siRNA (Materials and Methods). Following 24 hrs, supernatant p24 levels were measured and mean ± standard error values are graphically represented (D). Western blot analysis of Nedd4 protein levels in 293 cells transfected with HXB2, HXB2/Nedd4± siRNA is shown (E). Statistical significance, using a one way ANOVA followed by Tukey’s multiple comparison test, was shown for the decreased supernatant p24 levels from cells co-transfected with HXB2/siRNA (p<0.05) but not HXB2/control siRNA (p>0.05) as compared to cells with HXB2 alone.

In order to identify the protein domains that may be critical for the Nedd4-mediated increase in p24 levels, 293 cells were co-transfected with HXB2 and various Nedd4 protein domain mutants. Nedd4 is comprised of several protein domains including a Ca^2+^/calmodulin-regulated phospholipid binding domain (C2), 3–4 WW protein binding domains (WW1–4) and an ubiquitin ligase enzymatic (HECT) domain (Figures 3A, 4A). Initially, co-transfection of 293 cells was performed with HXB2 and plasmid DNA expressing either wild type rat Nedd4 (rNedd4) or a C/S mutant Nedd4 (rNedd4CSmut) that lacks ubiquitin ligase activity (Figure 3A). As shown in Figure 3B, transfection with either HXB2/rNedd4 or HXB2/rNedd4CSmut was found to yield significantly increased supernatant p24 levels at 48 hr (p<0.01) compared to levels obtained when cells were transfected with HXB2 alone. Supernatant p24 levels were not found to be statistically different (p>0.05) when comparing HXB2/rNedd4 and HXB2/rNedd4CSmut cultures. Western blot analyses demonstrated a low but detectable level of endogenous Nedd4 protein (Figure 3C). Nedd4 protein bands were markedly enhanced following rNedd4 and rNedd4CSmut transfection. These findings suggest that the mechanism by which Nedd4 is mediating an increase in p24 levels is not dependent on ubiquitin ligase activity.

Transfection studies were next performed using HXB2 co-expressed with either human Nedd4 (hNedd4), human Nedd4 lacking the C2 domain (hNedd4C2mut) or a specific splice variant of Nedd4-2 that lacks the C2 domain (Figure 4A). Consistent with the transfection studies described above, using rNedd4, exogenous co-expression of HXB2 and hNedd4 was found to significantly (p<0.01) increase supernatant p24 compared to cells transfected with HXB2 alone (Figure 4B). By contrast, transfection of 293 cells with hNedd4C2mut or hNedd4-2 did not result in a statistically significant (p>0.05) increase of p24 levels compared to HXB2 alone. Taken together, these findings suggest that the increase in supernatant p24 levels, by Nedd4, is dependent on the Ca^2+^/calmodulin regulated phospholipid binding C2 domain and not ubiquitin ligase activity.

### Nedd4 siRNA Reduces Supernatant p24 Levels in HXB2-Transfected Cells

Given that Nedd4/HXB2 co-expression in 293 cells demonstrated increased p24 levels (Figures 2–3, 4B), we hypothesized that inhibiting endogenous levels of Nedd4 may result in decreased levels of secreted p24. Hence, we co-transfected 293 cells with HXB2 and either a Nedd4-targeted siRNA previously shown to inhibit Nedd4 expression, or an irrelevant control siRNA targeting GFP [48]. When cell supernatants from these transfections were assayed for p24, levels were found to be markedly reduced in HXB2/siRNA-transfected cells as compared to HXB2-transfected cultures (Figure 4D). In contrast, p24 levels remained unchanged in samples where co-transfection included HXB2 and a control siRNA. While western blot analyses demonstrated that Nedd4-targeted siRNA could reduce endogenous Nedd4 protein levels, a more pronounced reduction was evident when cells were co-transfected with Nedd4 and siRNA compared to Nedd4 alone (Figure 4E).

### Exogenous Nedd4 Does Not Affect LTR Promoter Activity

**Figure 5:**
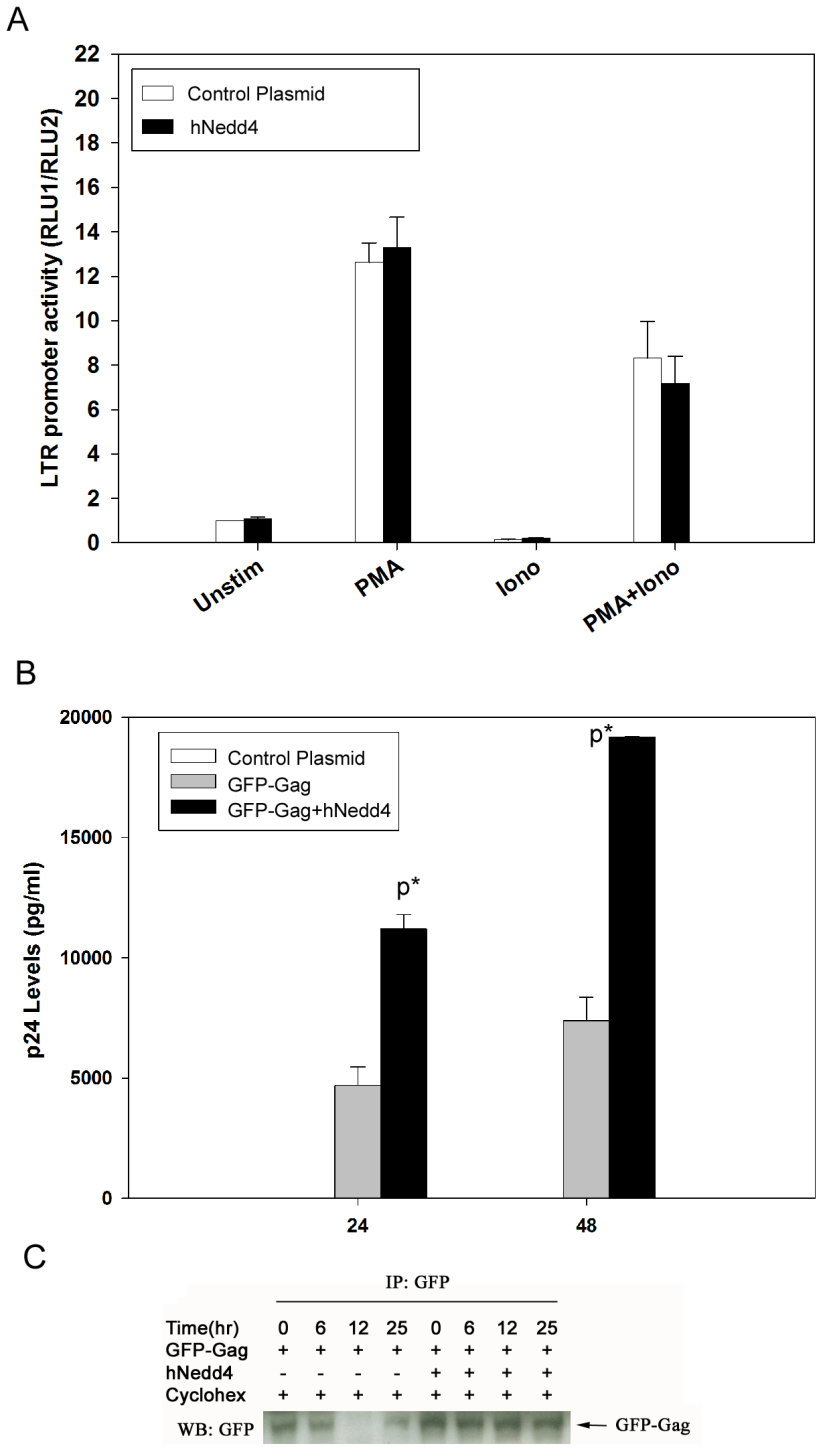
Nedd4 expression stabilizes intracellular HIV-1 protein levels but does not increase LTR promoter activity. Jurkat T cells were transfected with LTR-luciferase and pRL-TK-renilla reporter constructs in the absence or presence of hNedd4 (A). At 24 hr post-transfection, cells were either left unstimulated or stimulated with PMA, ionomycin (Iono) or with PMA+Iono. Jurkat T cells were transfected with LTR-luciferase and pRL-TK-renilla reporter constructs in the absence or presence of hNedd4. At 24 hr post-transfection, cells were either left unstimulated or stimulated with PMA, ionomycin (Iono) or with PMA+Iono. A dual luciferase assay was performed as per the manufacturer’s protocol (Materials and Methods) and mean ± standard error values for n = 3 experiments are shown graphically (A). No statistical significance, using a one way ANOVA followed by Tukey’s multiple comparison test, was shown for the supernatant LTR activity from cells co-transfected in the absence or presence of hNedd4 (p>0.05). Alternatively, 293 cells were transiently transfected with a GFP-Gag fusion construct in the absence or presence of hNedd4 (B). At 24 hr and 48 hr post-transfection, p24 assays were performed as described (Experimental Procedures). Mean p24± standard error values (n = 2) were calculated, graphically represented and statistical significance was shown for GFP-Gag/hNedd4 versus GFP-Gag (at 24 hr, 48 hr: p<0.05). 293 cells were transiently transfected with a GFP-Gag fusion construct in the absence or presence of hNedd4 were treated with cycloheximide at 24 hr post-transfection. Immunoprecipitations were performed using a rabbit anti-GFP polyclonal antibody followed by immunoblotting using a mouse anti-GFP monoclonal antibody on detergent-soluble cell fractions at 0, 6, 12, and 25 hr post-cycloheximide treatment (C).

We next tested the possibility that the Nedd4-mediated increase in p24 levels was driven by an increase in HIV-1 promoter activity. To this end, Jurkat T cells were transfected with an HIV-1 LTR-luciferase reporter construct in the absence or presence of hNedd4. At 24 hr post-transfection, cells were either left unstimulated or treated with phorbol ester [phorbol 12-myristate-13-acetate (PMA)], calcium ionophore [ionomycin (Iono)] or both PMA and Iono. In all treatment conditions tested, HIV-1 LTR activity remained unchanged by the presence of hNedd4 expression (Figure 5A).

### Nedd4 Mediates Increased p24 Levels in Gag-Transfected 293 Cells

We next investigated whether Nedd4 could mediate an increase in extracellular p24 levels when HXB2 was replaced with a plasmid DNA expressing Gag in co-transfection experiments. Hence, 293 cells were transfected with a GFP-Gag fusion construct in the presence and absence of hNedd4 and supernatant p24 levels were measured as described (Materials and Methods). Consistent with data shown with HXB2 (Figures 3–4), hNedd4 was found to significantly (p<0.01) increase supernatant p24 levels when cells were co-transfected with GFP-Gag compared to cells transfected with GFP-Gag alone (Figure 5B). This was noted both at 24 hr and 48 hr post-transfection.

### De Novo Protein Synthesis is Not Required for Nedd4-Mediated Increase in p24 Levels

In an effort to further understand the mechanism by which Nedd4 increases HIV-1 p24, we asked whether de novo protein synthesis is required for this modulation. A preliminary experiment was performed in which 293 cells were transfected with GFP-Gag in the absence or presence of hNedd4 for 24 hours followed by treatment with protein synthesis inhibitor, cycloheximide. Levels of Gag protein were then measured, by western blot analysis, at 0, 6, 12 and 25 hr post-cycloheximide treatment (Figure 5C). While Gag protein levels were found to decline over time following cycloheximide addition in the absence of ectopic hNedd4 expression, Gag remained elevated and did not decline in hNedd4-transfected samples (Figure 5C). Our data supports that idea that Nedd4 does not require de novo protein synthsis to mediate increased levels of HIV-1 p24.

### Nedd4 Co-Adminstration of BALB/c mice Yields Differential Vaccine-Specific Humoral and Cellular Immune Responses

**Figure 6:**
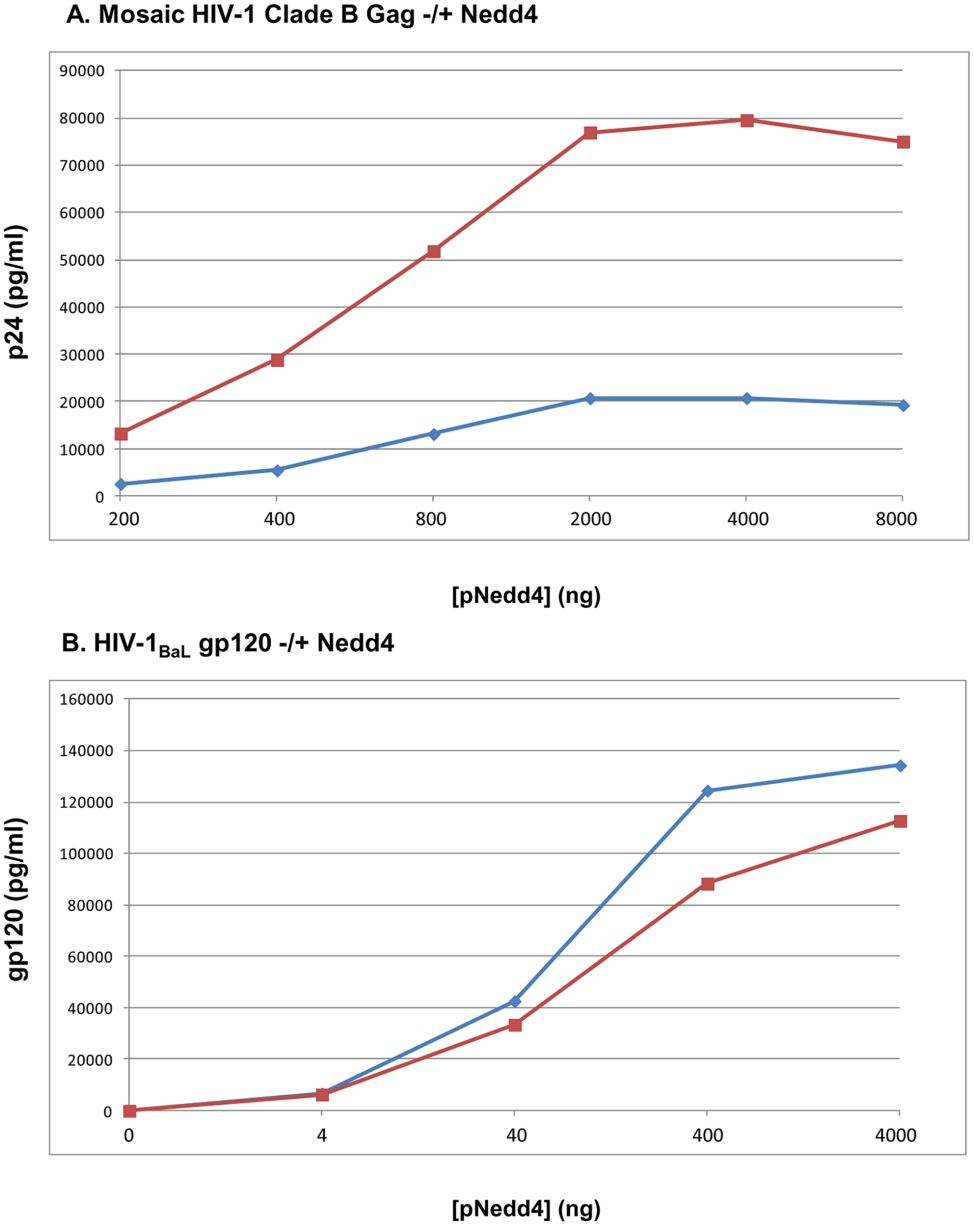
Increased Levels of Extracellular and Intracellular p24 Gag and gp120 Env Proteins by Nedd4. 293 cells were transiently transfected with a plasmid DNA expressing a clade B Mosaic Gag (A) or HIV-1_BaL_ gp120 (B) and increasing concentrations of hNedd4 as described (Materials and Methods). At 48 hr post-transfection, cell supernatants or cell lysates were assayed for p24 (A) or gp120 (B) by ELISA. Levels (pg/ml) of p24 (A) and gp120 (B) are represented graphically for supernatants (blue line) or cell lysates (red line).

Given the ability of Nedd4 to augment both intracellular and secreted HIV-1 p24 and gp120, we hypothesized that such increases could lead to augmented humoral and cellular immune responses following co-injection of plasmid DNA expressing Nedd4 and HIV antigens to BALB/c mice. Prior to immunizing mice, 293 cells were transiently transfected with plasmid DNA expressing HIV-1 *gag* or *env* and increasing concentrations of hNedd4. At 48 hours post-transfection, the levels of p24 and gp120 were assayed, by ELISA, using lysed cell pellets and cell supernatants (Figure 6A, Figure 6B). A Nedd4 dose-dependent increase in supernatant p24, intracellular p24, supernatant gp120 and intracellular gp120/gp160 was noted in these transfections.

**Figure 7:**
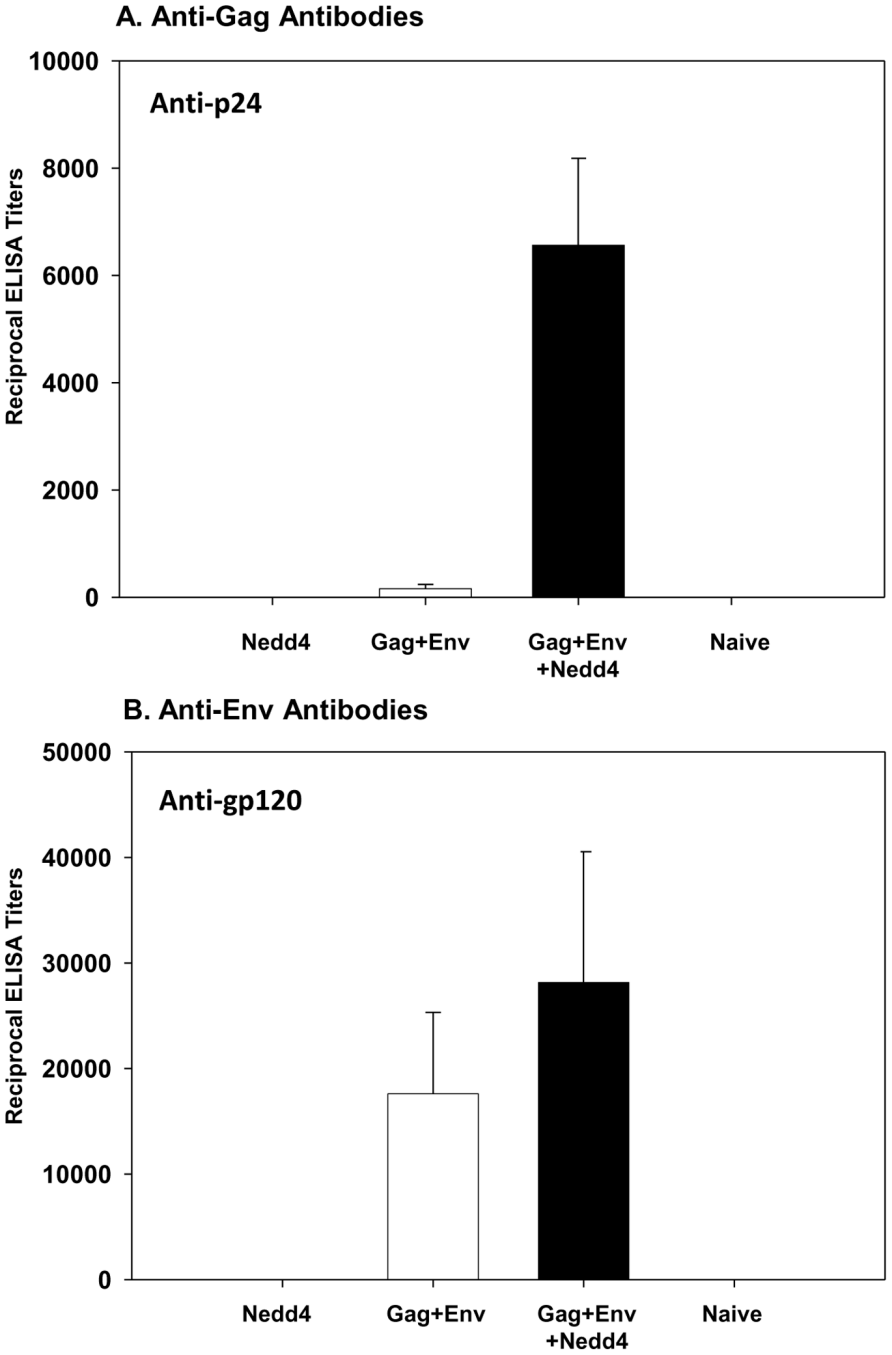
Differential Effects on Vaccine-Specific Humoral Immune Responses by Nedd4 Co-Adminstration of BALB/c mice. Intramuscular immunization of BALB/c mice (n = 5 per group; 5–6 week old females) was performed at weeks 0, 2 and 4 with plasmid DNA expressing human Nedd4 alone (Group 1), HIV-1 gag +env (Group 2) or human Nedd4+ HIV-1 gag+env (Group 3) as described (Materials and Methods). Group 4 mice (n = 3) were left un-immunized and served as naïve controls. Mice were sacrificed at week 6 to evaluate serum antibody responses (A, B). Vaccine-induced anti-p24 (A) and anti-gp120 (B) antibody levels were quantitated and reciprocal mean ELISA titers ± standard errors are graphically depicted. Statistical significance, using the Kruskal-Wallis nonparametric test followed by Dunn’s multiple comparison test, was noted in anti-p24 titers (Gag+Env+Nedd4 vs Gag+Env; P<0.05) but not anti-pg120 titers (Gag+Env vs Gag+Env+Nedd4: p>0.05).

**Figure 8:**
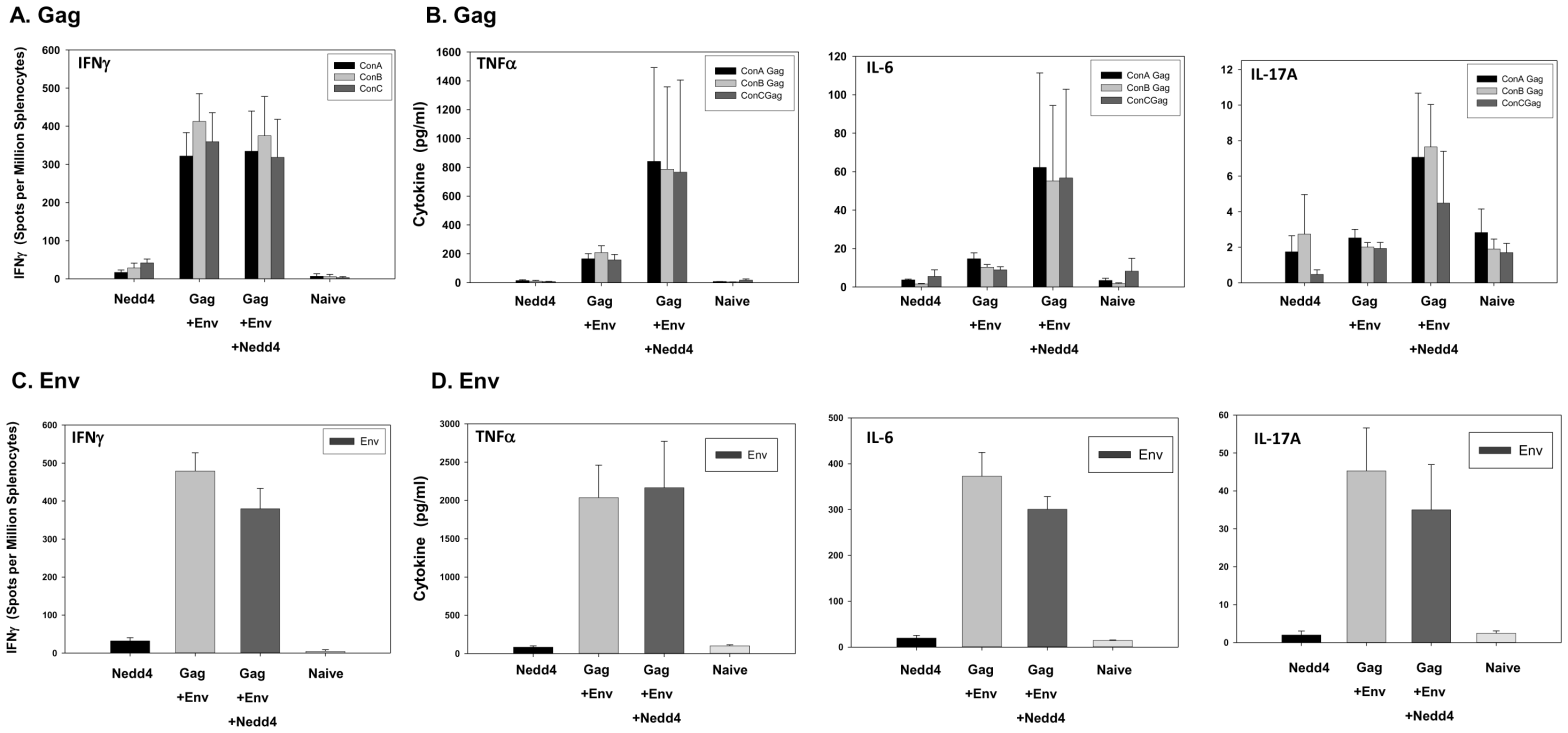
Effects of Nedd4 Co-Adminstration on Vaccine-Specific Cellular Immune Responses in BALB/c mice. Intramuscular immunization of BALB/c mice (n = 5 per group; 5–6 week old females) was performed at weeks 0, 2 and 4 with plasmid DNA expressing human Nedd4 alone (Group 1), HIV-1 gag +env (Group 2) or human Nedd4+ HIV-1 gag+env (Group 3) as described (Materials and Methods). Group 4 mice (n = 3) were left un-immunized and served as naïve controls. Mice were sacrificed at week 6 to evaluate splenocyte T-cell responses. Gag (A) and Env (C) peptide (consensus A, consensus B, consensus C)-specific IFNγ production was quantified by ELISPOT assay and mean spots per million splenocyte values ± standard errors are graphically represented. Gag peptide-specific TNFα (B, left panel), IL-6 (B, middle panel), IL-17A (B, right panel) and Env peptide specific TNFα (D, left panel), IL-6 (D, middle panel), IL-17A (D, right panel) cytokines were quantified by CBA from the supernatants of peptide stimulated splenocytes and are represented graphically as mean pg/ml values ± standard error. Statistical significance was assessed using the Kruskal-Wallis nonparametric test followed by Dunn’s multiple comparison test. No statistical difference in IFNγ, TNFα, IL-6 and IL-17A and values were found (Gag+Env vs Gag+Env+Nedd4: p>0.05).

BALB/c mice (n = 5 per group; 5–6 week old females) were then immunized intramuscularly at weeks 0, 2 and 4 with plasmid DNA expressing hNedd4 alone (Group 1), HIV-1 gag+env (Group 2) or hNedd4+HIV-1 gag+env (Group 3) as described (Materials and Methods). Group 4 mice (n = 3) were left un-immunized and served as naïve controls. Mice were sacrificed at week 6 to evaluate serum antibody responses and splenocyte T-cell responses. Nedd4 co-administration was found to increase vaccine-induced anti-p24 (Figure 7A; p<0.05). While there was a trend of Nedd4-mediated increases in anti-gp120 (Figure 7B; p>0.05), this observation was not found to be statistically significant. In order to test the hypothesis that hNedd4 co-administration could increase Gag- and/or Env-specific Th1/Th2/Th17 responses, we assessed peptide-specific IFNγ, TNFα, IL-2, IL-4, IL-6 and IL-17 cytokine levels following *in vitro* peptide stimulation of splenocytes. Gag (consensus A, consensus B, consensus C) and Env peptide-specific IFNγ production, quantified by ELISPOT assay, was not found to be significantly modulated by hNedd4 co-administration (Figure 8A, Figure 8C). Nedd4 also did not significantly alter the levels of Gag- or Env-specific IL-2 and IL-4, as measured by CBA (data not shown). Although not statistically significant (p = 0.6905–0.8413), a trend of increased Gag-specific TNFα (Figure 8B, left panel), IL-6 (Figure 8B, middle panel), and IL-17A (Figure 8B, right panel) was noted with hNedd4 co-administration. This trend, however, was not noted for Env-peptide specific cytokine responses in T-cells (Figure 8D).

## Discussion

It has now been demonstrated by a number of laboratories that HIV-1 exploits intracellular molecules to facilitate its trafficking through and budding from infected host cells [2], [5], [49]–[51]. Not surprising, it has also been shown that HIV-1 does not simply rely upon one molecule such as Tsg101 to bud, but it appears that the virus has evolved to make use of several intracellular proteins including AIP1/ALIX [1]. Given that HIV-1 Gag is post-translationally ubiquitinated and that a role for Nedd4 in HIV-1 trafficking and budding has not been fully explored, we investigated a potential role for this E3 ubiquitin ligase in HIV infection and pathogenesis. We hypothesized that if Nedd4 plays a key role in HIV pathogenesis, induction of Nedd4 would be observed post-infection of rhesus macaques with Simian-Human Immunodeficiency Virus and/or Simian Immunodeficiency Virus. Consistent with a role for Nedd4 in HIV infection, intra-rectal challenge of rhesus macaques with SHIV_SF162P3_ demonstrated an increase in Nedd4 protein post-infection (Figure 1). It is possible that HIV may be exploiting E3 ligases to facilitate virus replication and/or budding. Alternatively, we cannot rule out the possibility that early induction of Nedd4 and other E3 ligases represents, in-part, a host antiviral response that is exploited by the virus to mediate increased replication, budding and infection. This idea is consistent with the noted increase in Nedd4, by day 7 post infection (Figure 1E) that precedes peak viremia noted at day 14 (Figure 1A). We speculate that the subsequent decline in Nedd4 protein levels, by day 21, may reflect induction of a negative signaling pathway or cell death. A second round of increased Nedd4 levels, by day 42, may signal another host-antiviral response. Ongoing studies seek to understand the pathways involved in such Nedd4 modulation and if other E3 ligases are induced post infection with HIV/SIV/SHIV.

While Nedd4 was shown to augment intracellular and supernatant p24 levels in HXB2-transfected 293 cells, the increase was not dependent on ubiquitin ligase activity but rather on the Ca^2+^/calmodulin regulated phospholipid binding C2 domain (Figures 2–4). We therefore speculated that Nedd4 may be either functioning to increase viral expression, via augmented long terminal repeat (LTR) activity, or serving as an adapter to stabilize p24 protein levels and facilitate HIV-1 egress. While ectopic expression of Nedd4 did not affect LTR activity, (Figure 5A) transfection studies using Nedd4/Gag in conjunction with the protein synthesis inhibitor, cycloheximide, revealed a sustained level of Gag post-cycloheximide treatment that was not seen in the absence of Nedd4 (Figure 5C). Such findings are consistent with Nedd4 playing a role in stabilizing HIV-1 p24 and facilitating viral budding. More studies are needed, however, to assess the ability of Nedd4 to interact with and stabilize other HIV-1 proteins including gp160 or gp120. Previous work has shown that uncleaved gp160 is processed through lysosomes and only a small percentage of gp160 is cleaved to form the mature gp120 [52]. Hence, it is possible that Nedd4 could also play a role in the processing of the HIV envelope.

Several laboratories have now clearly demonstrated that Tsg101 binds HIV-1 Gag and thereby plays a critical role in mediating HIV-1 viral budding through late endosomes [49], [50]. We therefore speculated that if Nedd4 functions as an adaptor in facilitating HIV-1 trafficking and egress, it may do so by binding Tsg101 and thus recruiting the Tsg101/Gag complex to late endosomes. Consistent with this hypothesis, preliminary co-immunoprecipitation studies found an association of Tsg101 and Nedd4 (data not shown). While additional studies are needed to confirm this association, our findings suggest that HIV exploits Tsg101 and Nedd4 for trafficking and egress from target cells. HIV-1 has been shown to use different proteins to route the virus through different budding compartments [50], [53]. Studies to evaluate these pathways have demonstrated that HIV-1 budding can occur in primary macrophages and monocytic cell lines principally via late endosomes/MVBs [54], [55] and in T cells via the plasma membrane [53]. In our laboratories, Nedd4-mediated increased p24 levels were found to be similar in both T cell (H9, CEM) and monocytic (THP-1, U937) cell lines tested (data not shown). Our data suggests that Nedd4-mediated egress can occur via either late endosomes/MVBs or the plasma membrane.

Given that Nedd4 was shown to enhance intracellular and extracellular p24 Gag and gp120 Env, in co-transfection studies using plasmid DNA expressing either viral protein (Figure 6), we reasoned that Nedd4 co-administration to mice in the context of HIV vaccination could enhance vaccine-specific antibody and T-cell responses. Consistent with this hypothesis, Nedd4 co-injection in BALB/c mice was indeed able to augment anti-p24 antibodies (Figure 7A). Given the weak levels of anti-Gag antibodies versus robust levels of anti-Env antibodies (Figure 7B) noted following three DNA administrations of antigen alone, it is not surprising that a Nedd4-induced increase in humoral responses was only evident for anti-p24. Future studies will seek to lower the dose of plasmid DNA expressing *env* in an effort to assess if Nedd4 can mediate a dose sparing effect. While Nedd4 co-administration did not affect Gag- or Env-specific IFNγ T-cell responses (Figure 8A, Figure 8C), a trend of increased Gag-specific IL-6, IL17A and TNFα was noted (Figure 8B). Interestingly, the E3 ligase adaptor, Ndfip1, has been shown to regulate Th17 differentiation in mice [56]. Whether this increase in IL-6 and IL17A is a general function of E3 ligases or whether it is a Nedd4-specific phenomena remains to be determined. Given that the Nedd4-mediated increase in HIV-1 p24 was dependent on the C2 domain, future studies will focus on evaluating if increased humoral and cellular responses are noted in mice with co-administration of a plasmid DNA expressing the minimal C2 domain rather than the full Nedd4 sequence. Given that our findings showed differential effects, by Nedd4, on antibody and T-cell responses, more studies are needed to understand if such differences are due, in-part, to the use of separate plasmids for antigens and Nedd4 expression. Future studies will use a bi-cistronic plasmid to ensure that both antigens and Nedd4 are delivered to and expressed in the same cells. Overall, our findings that Nedd4 increases the levels of HIV proteins warrants further investigation into possible mechanisms of action as well as possible applications in the context of HIV vaccination.
